# Design and Optimization of Piezoresistive PEO/PEDOT:PSS Electrospun Nanofibers for Wearable Flex Sensors

**DOI:** 10.3390/nano10112166

**Published:** 2020-10-30

**Authors:** Eve Verpoorten, Giulia Massaglia, Gianluca Ciardelli, Candido Fabrizio Pirri, Marzia Quaglio

**Affiliations:** 1 Department of Applied Science and Technology, DISAT, Politecnico di Torino, 10129 Turin, Italy; giulia.massaglia@polito.it (G.M.); fabrizio.pirri@polito.it (C.F.P.); 2 Center for Sustainable Future Technologies, Italian Institute of Technology, 10144 Turin, Italy; 3 Department of Mechanical and Aerospace Engineering, Politecnico di Torino, 10129 Turin, Italy; gianluca.ciardelli@polito.it

**Keywords:** blend polymeric solution, electrospun PEDOT-PSS nanofibers, electrical conductivity, piezo-resistivity, flex mechanical sensor

## Abstract

Flexible strain sensors are fundamental devices for application in human body monitoring in areas ranging from health care to soft robotics. Stretchable piezoelectric strain sensors received an ever-increasing interest to design novel, robust and low-cost sensing units for these sensors, with intrinsically conductive polymers (ICPs) as leading materials. We investigated a sensitive element based on crosslinked electrospun nanofibers (NFs) directly collected and thermal treated on a flexible and biocompatible substrate of polydimethylsiloxane (PDMS). The nanostructured active layer based on a blend of poly(ethylene oxide) (PEO) and poly(3,4-ethylenedioxythiophene) doped with poly(styrene sulfonate) (PEDOT:PSS) as the ICP was optimized, especially in terms of the thermal treatment that promotes electrical conductivity through crosslinking of PEO and PSS, preserving the nanostructuration and optimizing the coupling between the sensitive layer and the substrate. We demonstrate that excellent properties can be obtained thanks to the nanostructured active materials. We analyzed the piezoresistive response of the sensor in both compression and traction modes, obtaining an increase in the electrical resistance up to 90%. The Gauge Factors (GFs) reflected the extraordinary piezoresistive behavior observed: 45.84 in traction and 208.55 in compression mode, which is much higher than the results presented in the literature for non-nanostructurated PEDOT.

## 1. Introduction

In the last decade, flexible and wearable electronic devices have gained increasing attention for application in several areas, ranging from healthcare and sports to space exploration and soft robotics [1,2,3]. Advancement in flexible sensors are largely responsible for the progress of wearable systems, and strain sensors received special attention by several application fields, such as health monitoring devices [4,5], artificial skin [6,7,8] and prosthetic limbs [9,10,11]. Efficient strain sensors have to be suitable for integration into flexible substrates and should change their electrical behavior in response to even small applied strains. Moreover, since in wearable applications strain sensors have to be interfaced with the human body, they must be highly stretchable and bendable in the general human perception range [4,5,6,7,8,9,10,11,12,13]. Strain sensors used in body joint monitoring are part of a subcategory of strain sensors named Flex Sensors. They sense the angular deformation rather than linear deformation [14]. Body joints monitoring plays an important role in several medical areas such as rehabilitation, diagnostic, fitness, as well as in ergonomics and for gesture modelling and recognition [14]. It is especially in rehabilitation that wearable sensors demonstrated to be potent tools able to overcome several issues potentially limiting the results achievable in traditional rehabilitation, such as the ability of the patient to perform the required tasks, the level of training and the experience of clinicians [15,16]. Rehabilitation of hand joints received particular attention since hand injuries are easily associated with disabilities that hinder peoples’ ability to carry out normal day-to-day tasks [17]. The demand for wearable, highly deformable, soft and light-weight flex sensors is ever increasing [18].

Materials selection plays a crucial role in designing sensors of this kind, not only to ensure the complex set of functional properties required by the final device, but also having to be biocompatible for interfacing with the human body. Nanomaterials have been established as the most promising candidates to satisfy this complex set of requirements and have been deeply investigated in the literature [2,8,9,11,12,13,19,20,21,22,23,24]. The development of nanostructures enhances the stimuli response of strain sensors working according to different sensing mechanisms, such as capacitance change [25,26], triboelectricity [27,28], piezoresistivity [5,29,30] and piezoelectricity [31,32], ensuring high sensitivity to the final devices. In terms of piezoelectric response, inorganic materials, such as ZnO nanowires [19], Pb(Zr,Ti)O_3_ (PZT) and BaTiO_3_ [33] were widely studied. To overcome the limits of these materials in terms of their processability, high production cost and low flexibility, polymeric materials received a considerable attention, especially when organized in nanostructures [34,35,36,37,38]. Polymer-based nanofibers, nanowires and composites nanomaterials, mostly based on graphene and carbon nanotubes [39,40,41,42,43,44,45,46,47], demonstrated excellent coupling of mechanical flexibility and electrical performance and have been widely employed as the functional material into strain sensors. Polyvinylidene fluoride (PVDF) is among the most frequently used polymer because of its intriguing piezoelectric properties that can be significantly enhanced by nanoconfinement [35] and the fact that it can be used in highly flexible and deformable polymer-based nanocomposite with good piezoelectric and piezoresistive features [48,49]. However, as reported in the literature [49], doping with nanofillers and nanoclays, embedded into nanoshaped PVDF, is frequently required to promote better performance for strain sensors.

Another important class of functional materials is based on intrinsically conductive polymers (ICPs). As reported by many works in the literature, ICPs present fantastic properties such as low cost, flexibility and above all, their versatility with many applications [50]. Among them, poly(3,4-ethylenedioxythiophene) (PEDOT), doped with poly(styrene sulfonate) (PEDOT:PSS) has been widely used due to its high stability, high electrical conductivity and excellent processability [50,51,52,53]. Further improvement of their properties can be obtained processing them into nanostructures, such as nanofibers by electrospinning [51]. In this way, the inherent properties of nanofibers, such as high porosity and high surface area to volume ratio can be exploited at their best to enhance the ICPs properties. In the present work, focus is given to the optimization of electrospun nanofibers (NFs) based on aqueous blends of polyethylene oxide and PEDOT:PSS (PEO/PEDOT:PSS) to develop sensitive nanomaterials that combine good electrical conductivity and good mechanical flexibility. As confirmed by many studies [51,52,54,55,56,57,58,59], PEDOT:PSS must be blended with other polymers to form electrospun nanofiber mats. To this purpose, several polymers have been employed, such as chitosan [54], polyvinyl alcohol (PVA) [55,56,57,58] and polyvinyl pyrrolidone (PVP) [59], but since they all have poor electric behavior the final nanofibers showed limited electrical conductivity. PEO is an interesting alternative because it is not only suitable to obtain a blend compatible to electrospinning, but it also shows amazing biocompatibility and it is also helpful to achieve excellent electrical conductivity of the final nanofiber mats [51,60]. PEO is able to promote crosslinking of the polymeric network, reacting with the PSS anion through thermal treatment under nitrogen atmosphere [61,62]. Optimal crosslinking is expected to improve the electrical conductivity, while preserving the mechanical properties of the nanofibers. High crosslinking degrees between PSS and PEO are responsible for an increase in the electrical conductivity of the final PEO/PEDOT:PSS NFs by the growth of the PEDOT domains. This growth promotes a rearrangement of PEO chains and improves the electron flow [61,63]. It has also been demonstrated that crosslinking in PEO/PEDOT:PSS NFs improves their water resistance, making them more versatile, and even suitable for applications, including placement of the sensitive nanofibers close to human body (i.e., skin) [51].

In the present work, piezoelectric stretchable PEO/PEDOT:PSS NFs were investigated and optimized as the sensing unit of flexible and wearable flex sensors. PEO/PEDOT:PSS NFs were directly deposited on a thin slab of polydimethylsiloxane (PDMS) that provided stability, flexibility and ease of application in contact to the human body. The thermal treatment (TT) of the PEO/PEDOT:PSS NFs was optimized, from a chemical point of view, monitoring (by infrared spectroscopy) the ethanol release as a product of the crosslinking reaction between PEO and PSS [64]. The analysis was performed in terms of the maximum applied temperature, the heating rate and the duration time of the overall treatment. The main purpose being to optimize the duration of TT with respect to the final electrical conductivity of the nanofibers, we introduced the ΔS/S_0_ parameter, which is the ratio of ΔS (i.e., the change in the electrical conductivity S_T_ due to the TT with respect to the starting one S_0_) over the electrical conductivity of the unprocessed nanofibers (S_0_).

Finally, the response of the PDMS-based flex sensors has been analyzed investigating the piezoresistive behavior of the crosslinked NFs mat. In order to demonstrate the great potential of the device for effective coupling to human body articulations, such as wrists, knees and elbows, the device has been tested inducing strain in both compression and traction mode by applying mechanical flexion. During strain application the electrical resistance R of the sensors has been measured, and the dimensionless parameter ΔR/R_0_ has been analyzed as a function of the applied deformation. ΔR/R_0_ represents the ratio of the change in the electrical resistance of the thermally treated sample (R_t_) and the untreated one (R_0_) over R_0_. A linear variation of the resistivity was obtained when the device was tested under compression, highlighting the elastic behavior of the nanofiber mat and underlying optimal recovery properties. The dimensionless resistance increased by 91% over a range of 160° in compression mode, which is a huge value in comparison to what can be found in literature [7,14,65,66]. Testing the device in traction mode evidenced a more complex behavior of the system: for large mechanical strain, the linear response of the sensor was limited by the plastic deformation of the nanofiber mats. However, this behavior does not limit the application of the sensor in the context of body joint monitoring, as we show in this work. Moreover, in traction mode, an increase of 90% of the resistance over a range of 90° was obtained, showing again the very good response of the system. The Gauge Factor was obtained through the model described by Saggio [67]. They were 45.84 in traction and 208.55 in compression mode. These results are much higher than the results presented in literature for non-nanostructurated PEDOT [7,53,65,68,69,70] showing the importance of the nanostructuration to improve the performances of flex sensors.

## 2. Material and Methods

### 2.1. Nanofibers Synthesis and Design of Flex Sensor

Polyethylene oxide (Mw = 600,000 Da) was purchased from Sigma-Aldrich (St. Louis, MO, USA) and PEDOT:PSS aqueous dispersion (PH1000) from Heraeus Clevios™ (Germany). The initial polymeric solution was based on (5 mL) of PEDOT:PSS added to a 5 wt% solution of PEO in deionized water, corresponding to a content of 4 wt% of PSS and 6.66 wt% of PEDOT with respect to PEO. The polymeric solution was aged overnight under stirring at room temperature. Nanofibers were obtained by electrospinning using a NANON 01A apparatus from MECC Ltd. (Japan). A high-power supply (HVU-30P100) powers a spinneret hosting a 27 Gauge × 15 mm needle. A syringe pump, connected to the spinneret, allowed selecting a flow rate in the range (0.1–99.9) mL/h. Each nanofiber mat was obtained by applying a voltage of 15 kV with a working distance of 15 cm for 15 min. A flow rate of 0.1 mL/h was selected to obtain an as uniform as possible distribution of the nanofiber diameters. Electrospun nanofibers were thermally treated at different heating temperatures, under inert atmosphere (360 sccm of N_2_ flow) with a heating rate of 2.5 °C/min, in a Carbolite, VST furnace from Nabertherm (Lilienthal, Germany), permitting final crosslinked nanofibers to be obtained, named in this work Crosslinked NFs. In particular, the heating treatment was conducted in two different steps: (i) the first one at 70 °C for 1 h under nitrogen atmosphere in order to preserve the morphology of the starting nanostructures; (ii) the second step is conducted at different temperatures and for different duration times. Furthermore, pure PEO NFs were applied as control nanomaterials. Nanofiber mats were directly deposited on a flexible substrate made of PDMS (purchased from Sylgard 184, St. Louis, MO, USA), prepared using a 10:1 weight ratio with respect to the curing agent and crosslinked at 100 °C to 1 h on a plate. In this way, we obtained binder free flex sensors, where Crosslinked NFs*,* representing the sensitive elements of the flex sensors*,* were directly integrated onto the flexible substrate of PDMS, which is able to confer stability and flexibility to the sensitive nanofiber mats. A paste made of PDMS, curing agent (10:1) and MWCNT (2 wt%) was spread at the two extremities of the nanofiber mat and cured at ambient temperature for one night. to create proper electrodes for electrical characterizations.

### 2.2. Morphological, Physical and Chemical Characterizations

#### 2.2.1. Field Emission Scanning Electron Microscopy

Field Emission Scanning Electron Microscopies (FESEM, Zeiss MERLIN, Oberkochen, Germany), operating from 5 to 10 kV were implemented to evaluate the morphological properties of Crosslinked NFs, in terms of preservation of the nanostructure morphology after crosslinking.

#### 2.2.2. Thermogravimetric Analysis Coupled with Fourier-Transform Infrared Spectroscopy

The crosslinking reaction and the corresponding thermal evolution during the TT was analyzed by thermogravimetric analysis (TGA), carried out using Perkin–Elmer Pyris 1 thermobalance (Waltham, MA, USA), under nitrogen atmosphere. To confirm the occurred crosslinking between PEO and PSS with the consequentl production of ethanol, TGA- FTIR measures were conducted coupling the Perkin–Elmer Pyris 1 thermobalance to a Perkin–Elmer Spectrum GX Infrared Spectrometer (Waltham, MA, USA). A Perkin–Elmer TG–IR ensured the coupling. To avoid the condensation of the reaction product while transferring from the TGA chamber to the IR unit and inside the unit itself, the Spectrometer was equipped of a gas cell heated at 220–230 °C.

#### 2.2.3. Electrical Characterizations

Moreover, electrical characterizations were performed with the purpose to analyze the electrical behavior of the Crosslinked NFs after the TT. A Keysight B2912A (Santa Rosa, CA, USA) source measure unit was used to perform I-V measurements. The voltage range applied was from −1 to 1 V, and the rate was 10 mV/s. The current flowing through the material because of its polarization was measured, and the resistance immediately interpolated. Three measurements were performed on each sample and in between these measurements, the sample went back to rest position. For the evaluation of the resistivity of the nanofibers by the second Ohm’s law, the thickness of nanofiber mats was measured by a surface profilometer (TENCOR P-10, Milpitas, CA, USA).

## 3. Results and Discussion

### 3.1. Fabrication of Flex Sensors with Sensitive Crosslinked NFs

**Figure 1:**
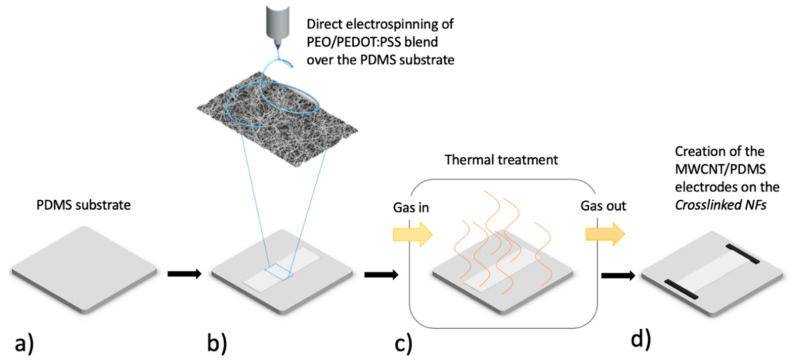
Schematic representation of the process flow used to fabricate the Crosslinked nanofibers (NFs) Flex Sensor starting from a PDMS substrate (**a**) by direct deposition of nanofiber mats by electrospinning using the PDMS substrate as the collector (**b**). The device is then thermally treated in controlled conditions to promote crosslinking into the PEO/PEDOT:PSS nanofibers and improve their electrical behavior (**c**). A PDMS/MWCNTs paste is spread at the edge of the Crosslinked NFs mat in order to obtain electrodes for electrical characterizations (**d**).

In this work, a binder free integration is proposed to couple a nanofiber-based sensitive piezoresistive material to a flexible substrate. It is based on the direct electrospinning of the PEO/PEDOT:PSS NFs on a PDMS substrate, as sketched in the process flow in Figure 1.

The PDMS substrate (Figure 1a) was selected to provide both stability and flexibility to the sensor and to offer surface features suitable for optimal mechanical coupling to the sensitive nanofiber mats. After electrospinning (Figure 1b), the flex system made by the substrate and the PEO/PEDOT:PSS NFs was thermally treated under controlled conditions, as shown in Figure 1c. The treatment was expected to promote crosslinking between PEO chains and PSS and to promote optimal adhesion between substrate and NFs. The active nanostructured layer after the treatment is referred to as Crosslinked NFs. Finally, as shown in Figure 1d, the electrical contacts were created by spreading a conductive paste obtained by mixing Multi-Walled Carbon Nanotubes (MWCNT) and PDMS (MWCNT/PDMS) at the extremities of the nanofibers mats and curing at ambient temperature for one night.

### 3.2. Optimization of Crosslinked Nanofibers Thermal Treatment

As the final aim of this work was the design of a novel, highly sensitive flex sensor based on nanofibers obtained from the blend PEO/PEDOT:PSS, the TT of the NFs was studied to identify the best conditions to:

(1) Preserve the nanostructuration of the electrospun nanofibers because this nanostructuration increases the performance of the sensor as demonstrated by Amjadi and coworkers [7];
(2) Promote the adhesion between the Crosslinked NFs and the PDMS substrate to obtain an optimal flex transfer;
(3) Activate the electrical conductivity of the functional material by crosslinking between PEO and PSS chains. To increase the performance of the sensor, we chose to integrate the nanofibers obtained by electrospinning in the sensor in a nanostructured form. However, to be electrospun, PEDOT:PSS must be blended [51,52,54,55,56,57,58,59]. In this work, PEO was chosen because, as Huang et al. coworkers showed, a significant improvement of the electrical conductivity of the blend, which is strictly related to the creation of crosslinks between PEO and PSS, is obtained. It is the creation of these links which promotes the growth of PEDOT domains [61,63].

**Figure 2:**
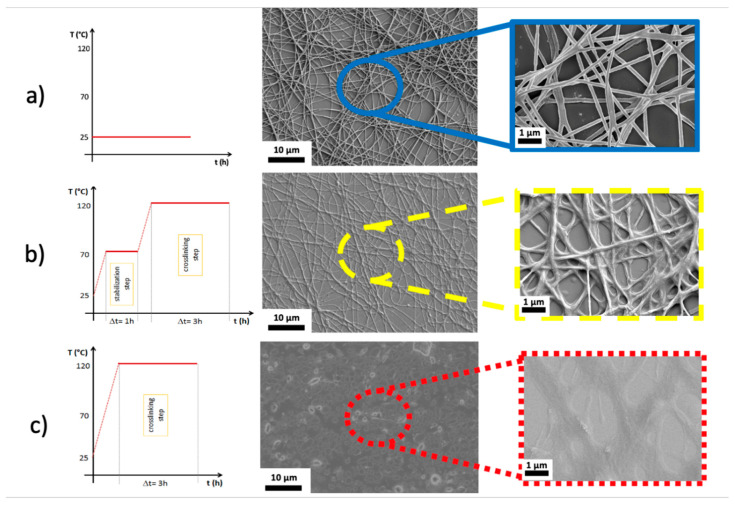
(**a**) morphology of no treated PEO/PEDOT:PSS NF mats (the blue (filled line) box is a magnification of FESEM images underlying the high surface area to volume ration and pore sized distribution); (**b**) morphology of Crosslinked NF mats, confirming the preservation of nanostructures as highlighted by the higher magnification reported by image in the yellow (dashed line) box; (**c**) morphology of Crosslinked NF mats but without the first step at 70 °C, showing destruction of nanostructures as highlighted by the higher magnification reported by image in the red (dotted line) box.

Different temperatures and duration times were valuated to obtain the optimal crosslinking process. In line with the results of Huang and coworkers [61], we were able to determine an ideal crosslinking temperature of 120 °C (see Supporting Information). However, for the flex sensor application, preserving the nanostructure of the Crosslinked NFs has crucial importance, and for that, a first stabilization step at 70 °C for one hour was implemented, as well as a slow heating rate of 2.5 °C/min to reach the steady states of the treatment. The temperature chosen was 70 °C because it is slightly higher than the melting temperature of PEO (67 °C), which allows the polymeric chains of PEO to reorganize themselves around PSS. FESEM images in Figure 2a–c show the nanofibers that were non-thermally treated, thermally treated with the full treatment and thermally treated at 120 °C for 3 h without the step at 70 °C, respectively. Figure 2b underlines the well-preserved nanostructures after heating treatment confirming no detrimental effects on the morphology and integrity of nanofibers as compared to no treated PEO/PEDOT:PSS NFs (as shown in Figure 2a). Moreover, Figure 2b highlights the pore distribution of Crosslinked NFs, whose dimensions are in the range of some micrometers. The pore distribution and size confirm the preservation of high surface area to volume ratio after heating treatment. However, Figure 2c clearly exposes how the nanostructure is destructed if the step at 70 °C is skipped. The diameter of the nanofiber-like structure on the red (dotted line) box of Figure 2c, which is definitively larger than the one of the Crosslinked NFs, shows a complete melting of the nanofibers into an almost continuous film, highlighting that avoiding the stabilization step hinders the preservation of the nanostructures. The step at 70 °C for one hour before the one at 120 °C is, therefore, essential, but also sufficient to conserve the properties of the nanofibers. Moreover, the partial melting of PEO leads to a perfect adhesion of the NFs mat to the PDMS support. As shown in Figure 2b, the Crosslinked NFs stick to the substrate and, therefore, ensure the mechanical strain transmission from the skin to the sensing element of the device—i.e., the NFs mat.

**Figure 3:**
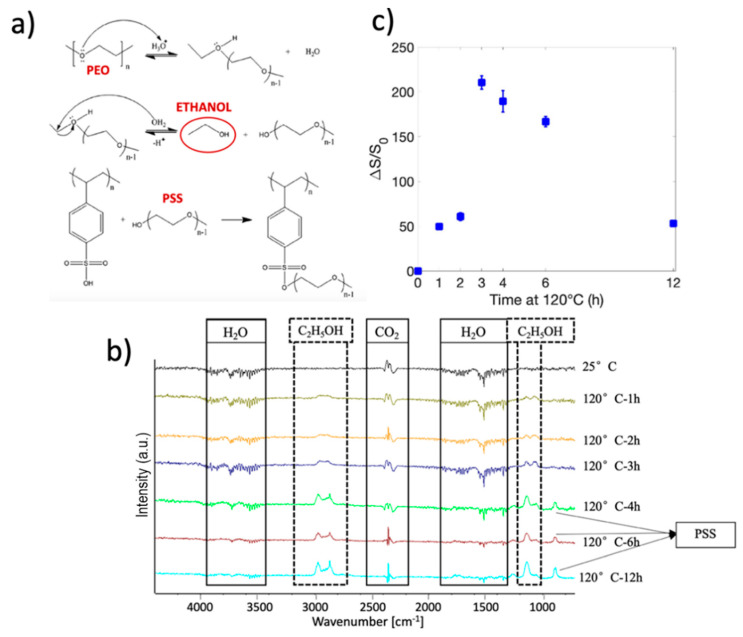
(**a**) Chemical scheme of the crosslinking reaction between PEO and PSS. Adapted from Polymer 54.23, Huang, T. M.; Batra, S.; Hu, J.; Miyoshi, T.; and Cakmak, M. Chemical cross-linking of conducting poly (3, 4-ethylenedioxythiophene): poly (styrenesulfonate) (PEDOT: PSS) using poly (ethylene oxide)(PEO), 6455–6462, (2013), with permission from Elsevier. (**b**) FTIR spectrum of the reaction products along the TT: 2.5 °C/min, 1 h @ 70 °C, xh @ 120 °C, at specific steps, under N_2_ atmosphere. (**c**) Electrical conductivity trend over the duration time of crosslinking reaction.

Huang and coworkers [61] demonstrated that the higher the links between PSS and PEO, the higher the electrical conductivity of PEO/PEDOT:PSS materials and evidenced that the crosslinking reaction produces ethanol (Figure 3a). FTIR analysis of the reaction products along the TT of nanofibers was performed to monitor the temperature at which crosslinking occurs. Figure 3b shows these results at pertinent steps on the TT. It is important to notice that since the measurement was conducted at ambient temperature and humidity, the peaks corresponding to water and CO_2_ are present in the results of the characterization. It is possible to appreciate the presence of the peaks associated with the release of ethanol (C_2_H_5_OH) starting from 120 °C. The presence of these peaks proves the occurrence of the crosslinking reaction between PSS and PEO. The details of the analysis of the peaks can be found in the supporting information.

From the hypothesis that the higher the links between PSS and PEO, the higher the electrical conductivity of Crosslinked NFs [61], we correlated the electrical conductivity variation with duration time of heating treatment (from a few minutes up to 12 h). We supposed that the longer the TT, the higher the number of links created. Figure 3c represents the trend of the ∆S/S_0_ parameter versus the duration time of the heating treatment. The electrical conductivity increases as the duration time increases, thus confirming the correlation between the electrical conductivity of the material and the crosslinking degree between PEO and PSS. In particular, it is evident that within the first three hours of TT, ∆S/S_0_ increases by more than two orders of magnitude and then decreases for TTs longer than 3 h. This trend can be attributed to the decomposition of PSS [64], as confirmed by the para-substituted benzene peak at 800–860 cm^-1^ that shows up after 3 h of TT at 120 °C in the FTIR spectra, as reported in Figure 3b. The degradation of PSS is expected to directly influence the electrical conductivity of the Crosslinked NFs because PSS degradation releases PEO chains, making them free to interfere with the electrons flow [61].

The optimized TT is, therefore, based on two steps: (i) the first one at 70 °C for 1 h, which is necessary to preserve the nanostructure and (ii) the second step conducted at 120 °C for 3 h to crosslink the polymers. Both the steps are performed under N_2_ atmosphere and with a heating rate of 2.5 °C/min. This preserves the nanostructuration of the electrospun nanofibers, promotes the adhesion between the Crosslinked NFs and the PDMS substrate to obtain an optimal strain transfer and activates the electrical conductivity of the functional material by crosslinking between PEO and PSS chains.

### 3.3. Characterization of Crosslinked NFs Flex Sensors

**Figure 4:**
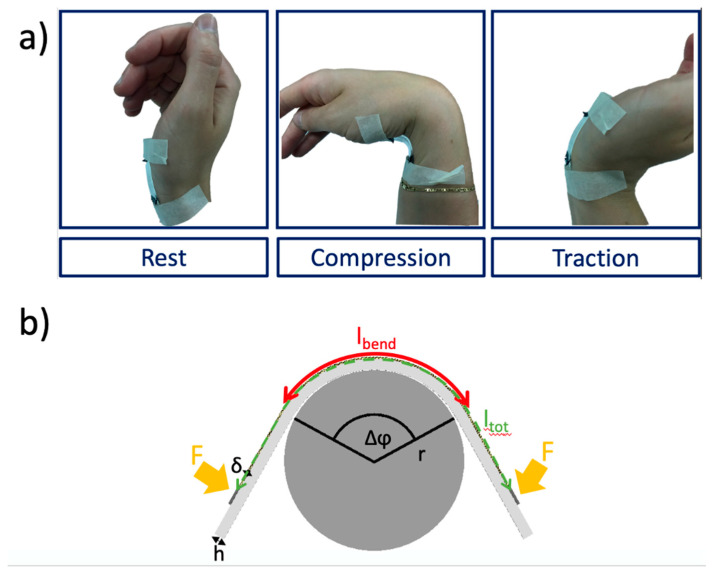
(**a**) Application of the sensor on the wrist. Rest, compression and traction modes illustrated. (**b**) Mechanical model of flex sensors used to obtain the Gauge Factor, following the paper of Giovanni Saggio [67].

With the aim to demonstrate the great potential of the devices for monitoring human body articulations, they were tested inducing strain in both compression and traction mode by applying mechanical flexion. An example of application is presented in Figure 4a, which shows the sensor placed in contact with the wrist. The biocompatible PDMS substrate ensures the optimal mechanical coupling to the body thanks to its spontaneous sticky behavior, and it offers at the same time optimal strain transfer to the active Crosslinked NFs layer. Medical tape further improves the sensor–skin mechanical coupling and especially helps to isolate the electrical contact from the skin. Three main working modes—i.e., rest, compression and traction—are illustrated from the left to the right, respectively, and it is easily seen that in traction mode the bending of the sensor does not exceed 90°. The good coupling of the sensor with the wrist, offered by the PDMS substrate, can be especially appreciated in both deformation modes.

**Figure 5:**
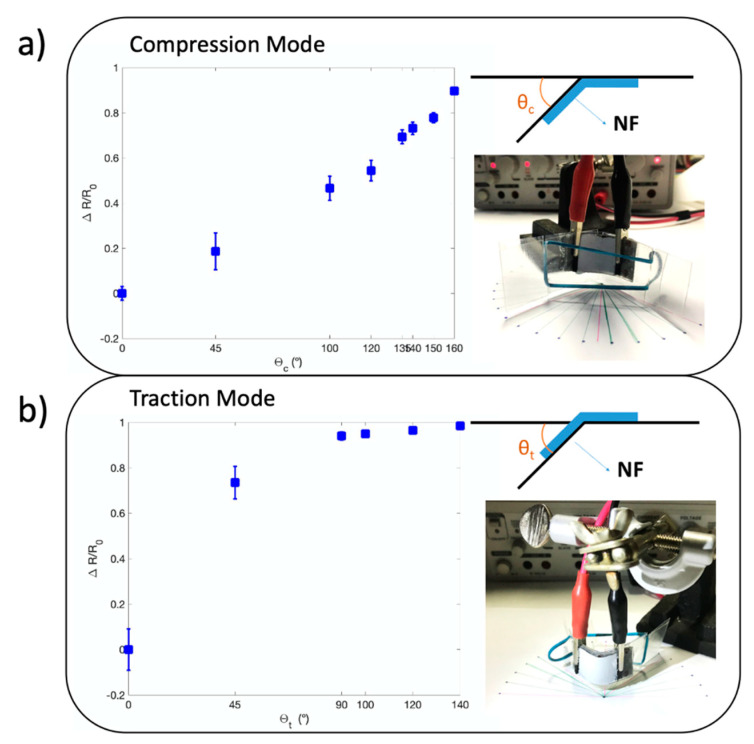
Resistance variation of the nanofibers mat when bending the samples: (**a**) compression mode highlighting an elastic behavior of Crosslinked NFs; (**b**) traction mode induced a plastic behavior of Crosslinked NFs. Inner schemes represent the definition of the angle θ in compression and traction modes. Inner images show the measurement set-up in both cases.

The correlation between the change in electrical resistance of Crosslinked NFs with the applied mechanical strain was analyzed by current–voltage measurements that were performed on the samples during bending at different angles, as shown in Figure 5. In particular, we introduce the $\Delta R/R_{0}$ parameter, which is the ratio between the resistance variation (ΔR), due to bending, to the resistance $R_{0}$ of the Crosslinked NFs when no deformation is applied. Figure 5a reports the results obtained when compression mode is applied to the sensors. In compression mode, a linear variation of Crosslinked NFs resistivity during the test is observed, highlighting an elastic behavior of nanofibers. An impressive increase in the resistance of the material up to 91% over a range of angular deformation of 160° was obtained, much higher than the $\Delta R/R_{0}$ value for a similar angular deformation range generally reported in literature [7,14,65,66].

In traction mode, as reported in Figure 5b, it is possible to see another huge resistance change of 90% over a range of 90°. However, a plastic behavior of nanofibers when the mechanical test is conducted at angular deformation higher than 90° is observed. Holding in consideration that almost all the body joints bend in one unique direction (knee, elbow, fingers, hip, ankle, etc.) or in the two directions but not exceeding 90° for one of the two (wrist), the plastic behavior of our sensor above 90° in traction mode is not considered as a problem. 

As Saggio showed [67], for a complete adhesion of the sensor to the skin and using the model described in Figure 4b, the Gauge Factor (GF) can be obtained directly by the following: $GF=\;\frac{\Delta R}{R_{0}}\cdot \frac{1}{\Delta \varphi }\cdot \frac{2l_{tot}}{h}$, with Δφ the angular deformation (in rad), $l_{tot}$ the length of the sensible component of the sensor (the NFs) and h the thickness of the PDMS substrate. In traction, we obtained a GF of 45.84 while in compression a GF of 208.55. The two values are very high in comparison with what can be found in literature [7] and confirm the sensitivity improvement through the nanostructuration and the crosslinking process of the material. PEDOT thin film-based sensors presented in literature have a GF up to 17.8 [7,65,68,69,70].

## 4. Conclusions

In the present work, we proposed an optimized sensitive element for flex sensors, based on Crosslinked NF mats, directly collected and thermal treated on a PDMS substrate. In particular, starting from an aqueous polymeric solution, based on a blend of PEO and intrinsically conductive PEDOT doped with PSS (PEDOT:PSS), we obtained PEO/PEDOT:PSS electrospun NFs. We investigated and optimized a thermal treatment with the aim to promote electrical conductivity of PEO/PEDOT:PSS NFs by crosslinking PEO and PSS, preserving the nanofibers’ morphology and optimizing the coupling between the sensitive layer and its substrate. We demonstrated that both the maximum temperature and the duration time of the heating treatment play a crucial role in determining an excellent electrical conductivity of the final samples. The optimal process is made up of two steps, a stabilization one at 70 °C for 1 h necessary to preserve the nanostructuration, followed by a step at 120 °C for 3 h under nitrogen atmosphere necessary for crosslinking. Thanks to the blending of PEDOT:PSS and PEO and through the crosslinking reaction, the electrical conductivity of the material was increased by two orders of magnitude. The final Crosslinked NFs mat showed high conductivity, high surface ratio to volume and high porosity. Moreover, the TT ensured a perfect adhesion of the sensing part of the device (the NFs mat) to the PDMS substrate, which allows an optimal mechanical strain transfer from the skin to the fibers. Finally, we investigated and confirmed the piezo-resistive response of the Crosslinked NFs Flex Sensors, demonstrating a linear variation of their resistivity during the compression mode test, highlighting an elastic behavior of nanofibers, without any hysteresis effect, underlying the ability of nanomaterials to recover the initial configuration after release of the mechanical strain. In compression mode, we obtained a huge increase of 91% in the resistance of the material in a 160° range. In traction mode, the resistance increase was of 90% over a range of 90°. Exceed this range led to a plastic behavior that, as we showed, is not a problem in the context of angular deformation of joints. The Gauge Factors obtained were 45.84 in traction and 208.55 in compression mode, which is much higher than the results presented in literature for non-nanostructured PEDOT. This shows the importance of the nanofiber form in our sensor.

## Supplementary Materials

The following are available online at https://www.mdpi.com/2079-4991/10/11/2166/s1, Figure S1: a: Weight percentage evolution and b: rate of degradation of PEO/PEDOT:PSS along temperature increase obtained by TGA analysis, Figure S2: FTIR spectrum of the reaction products along the thermal treatment: 2.5 °C/min, 1 h @ 70 °C, 2 h @ 120 °C, under N_2_ atmosphere. Above: thermal treatment of PEO nanofibers. Below: thermal treatment of PEO/PEDOT:PSS nanofibers, Figure S3: FTIR spectrum of the reaction products along the TT: 2.5 °C/min, 1 h @ 70 °C, x h @ 120 °C, under N_2_ atmosphere.
