# Supplementary material for: Design and Optimization of Piezoresistive PEO/PEDOT:PSS Electrospun Nanofibers for Wearable Flex Sensors
Source: Nanomaterials (Basel). 2020 Oct 30;10(11):2166. doi: 10.3390/nano10112166 (PMC7693578; doi:10.3390/nano10112166)
Supplement: Supplementary file 1 [file nanomaterials-10-02166-s001.pdf]

Supporting Information

Determining the temperature at which crosslinking occurs

To determine at which temperature the crosslinking occurs, thermogravimetric analysis (TGA) was conducted on the electrospun nanofibers. Weight percentage as well as degradation rate evolutions while the temperature increases are shown on **Error! Reference source not found.**a and S1b, respectively. Two deflections in the weight percentage variation and therefore two peaks in the degradation rate can be seen, corresponding to the two stages of deterioration that the material endures.

The first deflection peak starting at 100 °C was attributed to the ethanol produced by the crosslinking reaction.

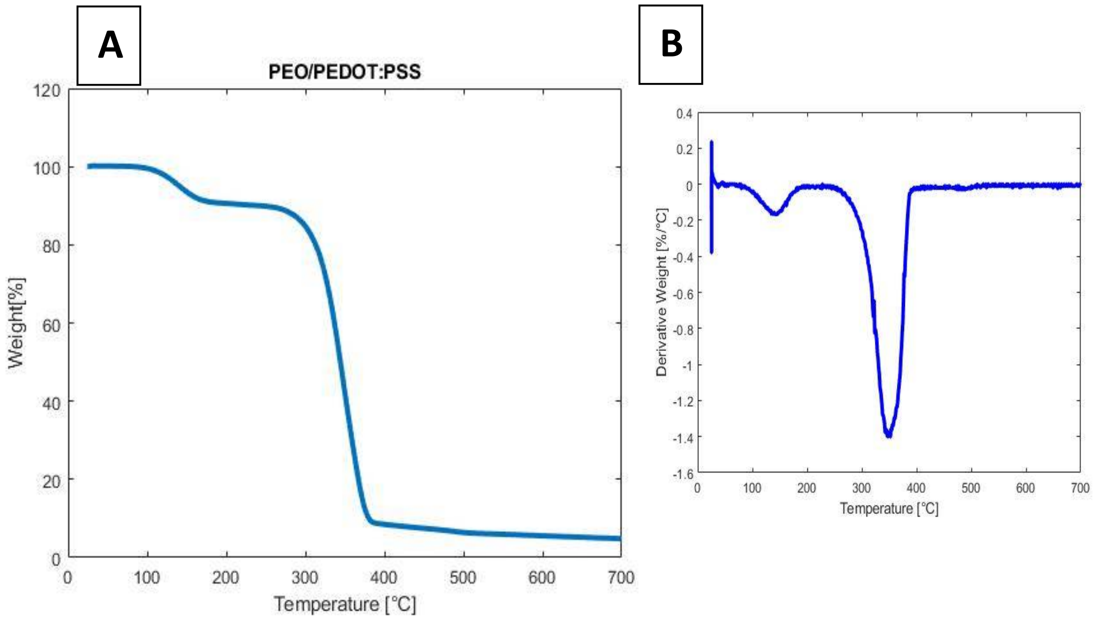

Figure S1 a: Weight percentage evolution and **b**: rate of degradation of PEO/PEDOT:PSS along temperature increase obtained by TGA analysis

To prove this statement, a Fourier transform infrared spectroscopy characterization was conducted on the reaction products along the thermal treatment of the fibers. This analysis was performed both on PEO and PEO/PEDOT:PSS nanofibers to be able to attribute the phenomenon observed at the presence of PEDOT:PSS on the fibers. The results are showed at **Error! Reference source not found.**2. The FTIR was performed on the gas that were extruded from the chamber containing the fibers when treated. Therefore, ambient parameters can influence the measure, and it is for that reason and for the huge adsorption capacity of PEO that we can see the peaks corresponding to water and CO_2.

However, at 120 °C, and only on the spectrum of PEO/PEDOT:PSS, CH_2, CH_3 and C-O peaks can be observed at 2850 and 2925 cm^{-1}, at 2870 cm^{-1} and 2960 cm^{-1}, and at 1048-1384 cm^{-1}, respectively. These peaks correspond to the production of ethanol, confirming that the crosslinking between PSS and PEO is occurring. The O-H peak at 3610–3670 cm^{-1} is not clearly observed because it is covered by the water production.

The second deflection starting at 300 °C can be directly attributed to the degradation of the components of the fibers since it was already proved that PSS degrades at 320 °C by the rupture of the sulfonate group from styrene, and PEDOT degrades at higher temperatures.[24] Moreover, the other TGA analysis on PEO nanofibers that were carried in the frame of this work could determine that PEO starts to degrade at 310 °C. Finally, this deflection leads to an almost complete loss of the material, supporting the hypothesis that it is provoked by the degradation of all the components togheter. Combining TGA and FTIR analysis, it could therefore be possible to identify the optimal temperature (120 °C) to allow the crosslinking reaction between PEO and PSS to occur. An additive step at 70 °C was added to the thermal treatment to ensure the evacuation of the remaining solvent present in the fibers.

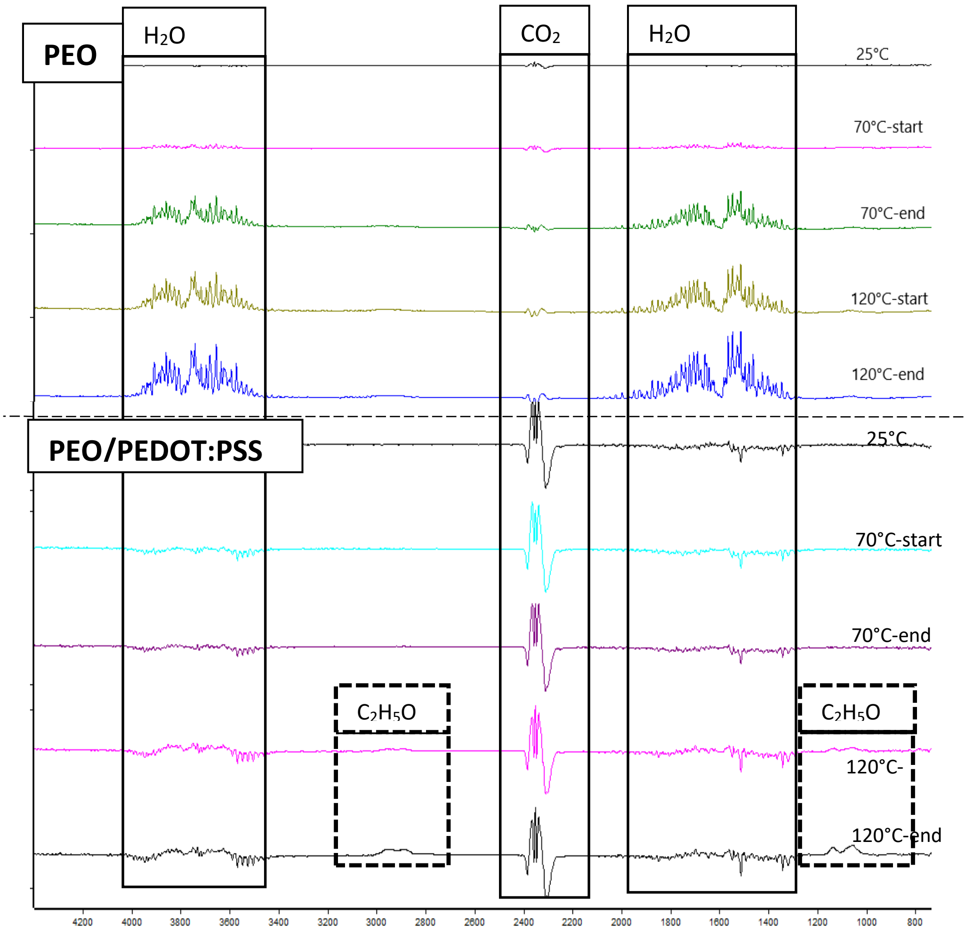

Figure S2: FTIR spectrum of the reaction products along the thermal treatment: 2.5 °C/min, 1 h @ 70 °C, 2 h @ 120°C, under N_2 atmosphere. Above: thermal treatment of PEO nanofibers. Below: thermal treatment of PEO/PEDOT:PSS nanofibers.

PSS degradation at crosslinking times higher than 3 h

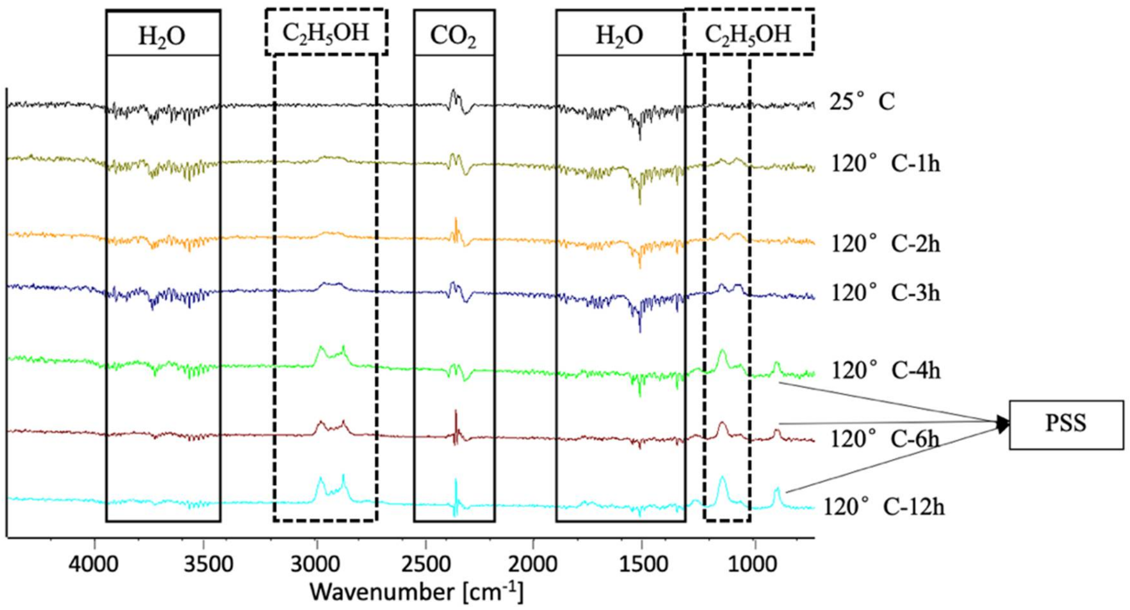

Figure S3: FTIR spectrum of the reaction products along the TT: 2.5 °C/min, 1 h @ 70 °C, x h @ 120 °C, under N2 atmosphere.

In the nanofibers, a decrease is observed when the thermal treatment is prolonged above 3 h. From the FTIR analysis conducted to understand this phenomenon (Figure S3), the decomposition of PSS is revealed by the para-disubstituted benzene peak at 800–860 cm^{-1} that shows up after 3 h of thermal treatment at 120 °C.
